# Patterns of lifestyle and their associations with anxiety symptoms among adolescents in Liaoning Province, China: a latent class analysis

**DOI:** 10.3389/fpubh.2025.1599214

**Published:** 2025-07-08

**Authors:** Qiyu Li, Le Chang, Liuyuan Li, Luyao Huang, Beibei Sun, Wenhui Fan, Shuhua Zhang

**Affiliations:** ^1^School of Medical Humanities, China Medical University, Shenyang, China; ^2^School of Educational Sciences, Shenyang Normal University, Shenyang, China

**Keywords:** latent class analysis, adolescents, lifestyle, anxiety, sleep quality

## Abstract

**Objective:**

To explore the latent categories of adolescents’ lifestyles and analyze the relationship between these lifestyle categories and anxiety.

**Study design:**

A cross-sectional survey.

**Methods:**

The questionnaire was designed to assess demographic characteristics, lifestyle behaviors, and anxiety. Sleep quality was measured by The Pittsburgh Sleep Quality Index (PSQI), anxiety symptoms were assessed by the Generalized Anxiety Disorder 7 (GAD-7), and lifestyle behaviors were operationalized as dietary behavior, physical activity, and sedentary time. Data collection was conducted from January to March 2024, it was employed to select adolescents from 12 cities in Liaoning Province. The lifestyles were classified using Latent Class Analysis (LCA), and an unordered multinomial logistic regression was performed to analyze the impact of different types of adolescent lifestyles on anxiety.

**Results:**

A total of 11,449 students were analyzed, and the prevalence of anxiety symptoms among adolescents is 32.62%. The participants were classified into three categories, High Sleep Diet - Low Activity (54.79%), Low Sleep Diet - Low Activity (9.01%), and High Sleep Diet - High Activity (36.20%). The results of unordered multinomial logistic regression showed that Age, gender, school location and lifestyle categories are significant factors influencing adolescent anxiety.

**Conclusion:**

Adolescents exhibit high levels of anxiety. Adolescents’ lifestyles can be categorized into three distinct groups. Lifestyle plays an important role in influencing anxiety. Schools, families, and society collaborate to implement effective intervention strategies, promoting healthier lifestyles to prevent and alleviate anxiety.

## 1 Introduction

Approximately 10 to 20% of adolescents globally experience mental health issues (1). Mental disorders have emerged as a global public health concern. A 2022 survey published in The Lancet Psychiatry reported that the global prevalence of anxiety was 3.1% (2, 3). Growing evidence suggests that mental disorders, such as anxiety, that emerge during adolescence may persist into adulthood (4, 5). In 2023, the National Health Commission of China, in collaboration with 17 other departments, issued the “Special Action Plan for Comprehensive Strengthening and Improving Mental Health Work for Students in the New Era (6)“. Emphasizing the need to enhance mental health initiatives for students and improve their mental health literacy (7).

Adolescence is a critical stage of rapid physiological and psychological development, during which individuals are particularly vulnerable to emotional problems such as anxiety and depression. Concurrently, significant shifts in adolescents’ lifestyles often occur, including alterations in sleep patterns, physical activity levels, and dietary habits (8). According to the 2022 China Youth Health Behavior Research, based on survey data from 10 provinces and cities, significant differences exist in the lifestyles of adolescents. Approximately one-quarter of the surveyed adolescents do not engage in physical exercise, and the average number of days per week that adolescents eat breakfast is 3.7 (6). Unhealthy lifestyles, including irregular eating habits, insufficient physical activity, and poor sleep patterns, are strongly associated with adolescents’ mental health (8–16). For instance, Smirnova et al. (17) found that poor sleep habits, increased screen time, and reduced physical activity exacerbate the development of anxiety symptoms. However, most previous studies have focused on examining the impact of a single lifestyle factor on anxiety (18–21), overlooking the role of various lifestyle combinations in adolescent anxiety.

An individual’s lifestyle typically consists of various behavioral habits, and different combinations of lifestyle factors may have varying degrees of impact on anxiety. Focusing solely on a single factor may not fully elucidate the complex relationship between lifestyle and anxiety. Cluster analysis is an effective method for classifying various factors into subgroups by identifying the similarities in individual characteristics. It can divide samples into multiple groups (or classes), thereby aiding in the understanding of the relationship between lifestyle and mental health. Numerous studies have successfully employed cluster analysis to identify specific lifestyle patterns and link them with mental health indicators, such as anxiety and depression. However, traditional cluster analysis faces challenges, including instability in classification and insufficient handling of data complexity. In particular, adolescent anxiety has become an increasingly severe issue in modern society, and the complexity and interrelation of their lifestyle behaviors make it difficult for a single analysis method to reveal its impact effectively. Because adolescent lifestyle behaviors are typically multidimensional and interrelated, Latent Class Analysis (LCA), as a more comprehensive and advanced statistical method, can identify latent subgroups within individuals, explore group heterogeneity, and handle complex data. For these reasons, LCA has been widely applied in areas such as disease symptom classification and behavioral pattern identification (14).

This study employs LCA to identify different adolescent lifestyle types, including physical activity, weekday sedentary behavior, weekend sedentary behavior, dietary habits, and sleep duration. It also examines the prevalence of anxiety symptoms among adolescents and investigates the relationship between different lifestyle categories and anxiety. We hypothesize that different combinations of lifestyle factors will have varying impacts on the occurrence of anxiety symptoms in adolescents. The study aims to provide guidance for schools, families, and society in developing more targeted mental health intervention strategies, assisting adolescents in establishing healthy lifestyles, and preventing or alleviating anxiety symptoms. The findings of this study offer valuable insights into global adolescent mental health strategies. In particular, considering the unique challenges faced by Chinese adolescents in lifestyle factors (such as irregular eating habits, lack of exercise, and sleep issues), the study provides theoretical support for developing comprehensive mental health interventions for adolescents worldwide and offers specific guidance for policymakers and practitioners.

## 2 Methods

### 2.1 Calculation of minimum sample size

The formula for sample size calculation is *n* = [Z_*α*/2_^2^pq]/δ^2^. In the formula, *n* symbolizes the sample size, and p is for the estimated prevalence of anxiety among adolescents, *q* = 1−*p*, *α* = 0.05, Z*α*/2 = 1.96 ≈ 2, and *δ* is the acceptable error (*δ* = 0.1*p). Based on the literature, the prevalence of anxiety among adolescents ranges from 13.9 to 36.15% (22–24). The minimum sample size obtained from the above prevalence rate was 2,380. Considering the possible 10–15% invalid questionnaire rate (25, 26), the minimum sample size obtained from further calculation was 2,618–2,737.

### 2.2 Setting and sampling

Between January and March 2024, students from grade 1 of junior high school to grade 3 of senior high school in 49 schools across 12 cities in Liaoning Province, China, were selected as study participants. After obtaining informed consent, students were asked to complete paper-based questionnaires on-site. Inclusion criteria: Health status: No severe cognitive impairments, able to understand and respond to the content of the questionnaire. Vision: No severe vision impairments, able to read the questionnaire or complete it using assistive devices. Consent to participate: Voluntary participation, with a signed informed consent form. Exclusion criteria: Limited physical activity: Individuals unable to engage in regular physical activities due to health or physical reasons. Absence from school: Students who were on leave or had suspended their studies for any reason.

### 2.3 Research instruments

#### 2.3.1 General characteristics of respondents

The demographic characteristics include gender, age, school, school location, and grade.

#### 2.3.2 Research instrument

##### 2.3.2.1 Sleep quality

The Pittsburgh Sleep Quality Index (PSQI) was used to assess the sleep quality of participants over the past month. The scale consists of 18 items, assessing factors such as sleep onset time, sleep duration, sleep efficiency, sleep disturbances, sleep quality, use of sleep medication, and daytime dysfunction. A higher score indicates poorer sleep quality, with scores of 1 and 2 corresponding to good and poor sleep quality, respectively. The PSQI has been validated in several studies, demonstrating good reliability (Cronbach’s *α* coefficients of 0.83 and 0.73) (27, 28).

##### 2.3.2.2 Dietary behavior

This questionnaire was developed by Li in 2022. It includes questions regarding whether participants eat meals on time, the importance of each meal, binge eating behaviors, the habit of eating meals or snacks while studying or watching TV, and the frequency of consuming breakfast, fruit, vegetables, and milk. It also assesses whether participants have access to desired foods based on current living conditions and their self-assessment of the healthiness of their eating behaviors. The questionnaire consists of 10 items, with a total score of 8 points. A score of 60% or above is considered “good” (scored as 1), and below 60% is considered “poor” (scored as 2) (29). The reliability of the scale is 0.722.

##### 2.3.2.3 Physical activity

Physical activity measurement is derived from the Health Behavior in School-aged Children (HBSC) survey. Since low-intensity physical activity does not provide significant health benefits, while moderate and vigorous activities offer more evident benefits, this study focuses specifically on moderate-to-vigorous physical activity. “Moderate-to-vigorous physical activity” is defined in the questionnaire as “any activity that increases the heart rate and leaves you out of breath for a period, including various physical activities during physical education classes, exercise, training, and daily life (e.g., hiking, outdoor activities, brisk walking).” The total number of days from both questions represents the participant’s weekly physical activity. According to the 2023 China Children and Adolescents Physical Activity Guidelines, engaging in physical activity for 7 days a week is considered healthy (scored as 1), whereas fewer than 7 days is considered unhealthy (scored as 2) (30).

##### 2.3.2.4 Sedentary time

The questionnaire was developed by Yang (31) based on the long version of the International Physical Activity Questionnaire (IPAQ), with adjustments and improvements made through consultations with experts in the research context. The formula for calculating daily sedentary time is as follows: Daily Sedentary Time = (Total sedentary time from Monday to Friday * 5 + Total sedentary time on the weekend * 2) / 7. According to the 2018 China Children and Adolescents Physical Activity Guidelines, sedentary time of ≥2 h is scored as 1 (unhealthy), while sedentary time of <2 h is scored as 2 (healthy).

#### 2.3.3 Anxiety

The Generalized Anxiety Disorder 7 scale (GAD-7), developed by Spitzer et al. (32) was used to assess the frequency of anxiety symptoms experienced by participants over the past 2 weeks. This scale consists of 7 items, each scored on a 4-point system (0 = “not at all” to 3 = “nearly every day”). The total score ranges from 0 to 21, with cutoff points of 5, 10, and 15 indicating mild, moderate, and severe anxiety, respectively. The total score is categorized as follows: 0–4 = no anxiety, 5–9 = mild anxiety, 10–14 = moderate anxiety, and ≥15 = severe anxiety. This scale has been validated in multiple studies as an effective tool for evaluating anxiety (33). The Cronbach’s *α* coefficient for this scale is 0.954.

### 2.4 Quality control

Before the start of the survey, this study trained the selected investigators on how to distribute questionnaires to respondents and registered the respondents’ codes face-to-face, one by one. Every Sunday evening, the research team members communicated with the investigators to summarize, evaluate, and provide feedback on the questionnaires they had collected. After the questionnaires were collected, two researchers conducted thorough logic checks and data screening. This study also carried out the cleaning of the missing data.

### 2.5 Statistical analyses

LCA (used LCA to test whether there are different potential categories of adolescent behavioral lifestyles) of adolescent behavioral lifestyles was performed using Mplus 8.3 software. The model fitting indices for the LCA included the Akaike Information Criteria (AIC), Bayesian Information Criteria (BIC), adjusted Bayesian Information Criteria (aBIC), Lo–Mendell–Rubin Likelihood Ratio Test (LMRT), Bootstrapped Likelihood Ratio Test (BLRT) and Entropy. Using R, the posterior probabilities for each individual’s latent class membership were calculated, along with the overall average posterior probability (AvePP) based on the highest posterior value per individual, to further assess classification accuracy. Based on the selected fit model, the latent classes of adolescent behavioral lifestyles were used to analyze the relationship between anxiety and the different behavioral lifestyle categories. Group comparisons were conducted using Kruskal-Wallis H (due to the data not conforming to a normal distribution) and Mann–Whitney U tests (for further pairwise comparisons to explore significant differences between specific groups). Unordered multinomial logistic regression (to explore the relationship between predictor variables and outcomes with more than two categories, addressing the non-ordinal nature of anxiety levels) was performed to analyze the impact of different types of adolescent lifestyles on anxiety.

## 3 Results

### 3.1 General information

The study included a total of 11,449 adolescents, aged 11 to 20 years, with an average age of 14.92 ± 1.59 years. Of these, 5,454 (47.6%) were male, and 5,995 (52.4%) were female. The schools were located in various cities across China: 1,926 cases (16.8%) in second-tier cities, 5,840 cases (51.0%) in fourth-tier cities, and 3,683 cases (32.2%) in fifth-tier cities.

### 3.2 Latent class model fit for adolescent behavioral lifestyles

This study explored the latent class model for adolescent behavioral lifestyles, with model fit presented in Table 1. The AIC, BIC, and aBIC values generally decreased as the number of classes increased. The *p*-values for the LMRT and BLRT were less than 0.001 for the 2-class to 4-class models, with an Entropy value of 0.675 for the 4-class model. The Entropy value reached a maximum of 0.832 for the 3-class model. In the 4-class model, the Entropy value was below 0.8. Based on a comprehensive evaluation of all indices, the 3-class model demonstrated the best fit; therefore, the 3-class model was selected. The specific details are provided in Table 1.

**Table 1:** Model fit indices for different latent classes.

Class	AIC	BIC	aBIC	*p*		Entropy	Class probability
				LMRT	BLRT		
1	61000.125	61036.853	61020.964	<0.001	<0.001	/	1.000
2	55997.573	56078.375	56043.418	<0.001	<0.001	0.784	0.737/0.263
3	55336.605	55461.481	55407.457	<0.001	<0.001	0.832	0.548/ 0.090/0.362
4	55100.836	55269.786	55196.695	<0.001	<0.001	0.675	0.347/0.0750.302/0.275
5	55105.176	55318.200	55226.041	0.063	0.188	0.726	0.034/0.260/0.375/0.280/0.051

### 3.3 Latent class characteristics of adolescent behavioral lifestyles

**Figure 1:**
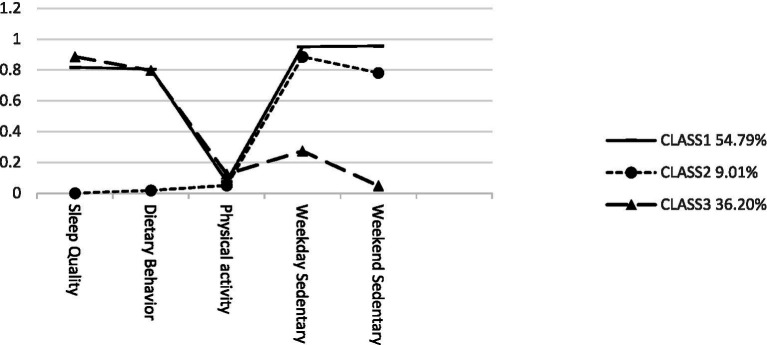
Conditional probability plot of adolescent behavioral lifestyle latent classes.

Adolescent behavioral lifestyles were divided into three latent classes in this study, as shown in Figure 1 and Table 2. Class 1 (*n* = 6,232, 54.79%): Key characteristics of this group include high sleep quality, a healthy diet, low physical activity, and the highest sedentary time during both weekdays and weekends. This group was labeled as the “High Sleep Diet- Low Activity Group.” Class 2 (*n* = 1,032, 9.01%): Key characteristics of this group include poor sleep quality, an unhealthy diet, the lowest physical activity, and relatively high sedentary time during both weekdays and weekends. This group was labeled as the “Low Sleep Diet - Low Activity Group.” Class 3 (*n* = 4,144, 36.20%): Key characteristics of this group include the highest sleep quality, a relatively healthy diet, relatively high physical activity, and lower sedentary time during both weekdays and weekends. This group was labeled as the “High Sleep Diet - High Activity Group.” Class 1 and Class 2 share similar probabilities for physical activity and sedentary behavior, whereas Class 1 and Class 3 share similar probabilities for sleep quality and dietary behaviors. The overall AvePP was 0.930, indicating a high degree of classification reliability. Specifically, the AvePP was 0.968 for Class 1, 0.745 for Class 2, and 0.919 for Class 3, suggesting that the classification precision was excellent for Classes 1 and 3, while Class 2 fell within an acceptable range. The specific details are provided in Figure 1 and Table 2.

**Table 2:** Naming of adolescent behavioral lifestyle latent classes and their conditional probabilities and class probabilities.

Item	Class	Class 1 (High sleep diet - Low activity)	Class 2 (Low sleep diet - Low activity)	Class 3 (High sleep diet - High activity)
Sleep quality	Good	0.817	0	0.886
	Bad	0.187	1	0.114
Diet behavior	Good	0.805	0.018	0.797
	Bad	0.195	0.982	0.203
Physical activity	Good	0.076	0.051	0.124
	Bad	0.924	0.949	0.874
Weekday sedentary	Long	0.951	0.886	0.274
	Short	0.049	0.114	0.726
Weekend sedentary	Long	0.956	0.781	0.048
	Short	0.044	0.219	0.952
Class probability	—	0.5479	0.0901	0.3620

— indicates no relevant data.

### 3.4 Univariate analysis of anxiety levels across demographic variables and behavioral lifestyle categories

The univariate analysis results indicated that gender, family location, school location, and behavioral lifestyle categories were significantly associated with anxiety levels (*p* < 0.05). Female adolescents exhibited significantly higher anxiety scores than males. Adolescents from second-tier cities had the highest anxiety scores, whereas those from fifth-tier cities had the lowest. The “Low Sleep Diet - Low Activity” group had the highest anxiety scores, while the “High Sleep Diet - High Activity” group exhibited the lowest anxiety scores. The details are provided in Table 3.

**Table 3:** Univariate analysis of anxiety in adolescents.

Item	Group	*N*	Anxiety	*Z/H*	*p*
		Rank Mean			
Gender				*Z* = −6.728	<0.001
	Male	5,454	5544.71		
	Female	5,995	5889.02		
School location				*H* = 97.834	<0.001
	Second-tier city①	1926	6228.99		
	Fourth-tier city	5,840	5525.53		
	Fifth-tier city	3,683	5777.73		
Behavioral lifestyle				*H* = 1195.882	<0.001
	High Sleep Diet- Low Activity	6,273	5517.87		
	Low Sleep Diet - Low Activity	1,032	8521.20		
	High Sleep Diet - High Activity	4,144	5342.20		

①This classification is based on the urban tier classification in China (50, 51).

### 3.5 Unordered multinomial logistic regression analysis of anxiety

An unordered multinomial logistic regression analysis was conducted, with anxiety (“no anxiety” as the reference category) as the dependent variable and four statistically significant variables from the univariate analysis—gender, age, school location, and latent lifestyle category—as independent variables. The results of the parallelism test indicated a *P* of < 0.05, supporting the use of an unordered multinomial logistic regression analysis.

The results of the regression analysis revealed the following: Age was significantly associated with mild anxiety (OR = 1.107, 95% CI = 1.073–1.142, *p* < 0.001) and severe anxiety (OR = 1.013, 95% CI = 1.032–1.179, *p* = 0.004) when compared with the “no anxiety” group. Males had a lower risk of mild anxiety (OR = 0.786, 95% CI = 0.715–0.865, *p* < 0.001), moderate anxiety (OR = 0.865, 95% CI = 0.750–0.998, *p* = 0.047), and severe anxiety (OR = 0.774, 95% CI = 0.635–0.945, *p* = 0.012) compared to females. Adolescents from second-tier cities had a higher risk of mild anxiety (OR = 1.717, 95% CI = 1.489–1.980, *p* < 0.001), moderate anxiety (OR = 1.469, 95% CI = 1.187–1.817, *p* < 0.001), and severe anxiety (OR = 1.848, 95% CI = 1.404–2.432, *p* < 0.001) compared with those from fifth-tier cities. Adolescents in the “Low Sleep Diet - Low Activity” group (OR = 1.333, 95% CI = 1.200–1.482, *p* < 0.001) had a higher risk of mild anxiety compared with the “High Sleep Diet - High Activity” group. Adolescents in the “Low Sleep Diet - Low Activity” group had significantly higher risks of moderate anxiety (OR = 11.044, 95% CI = 8.872–13.747, *p* < 0.001) and severe anxiety (OR = 16.031, 95% CI = 12.201–21.063, *p* < 0.001) compared with those in the “High Sleep Diet - High Activity” group. The details are provided in Table 4.

**Table 4:** Unordered multinomial logistic regression analysis of anxiety.

Anxiety Group	Variable	*B*	SE	*Wald χ^2^*	*p*	OR	95% CI
No anxiety vs. Mild anxiety	Intercept	−2.832	0.258	120.955	<0.001	—	—
	Age	0.101	0.016	40.441	<0.001	1.107	(1.073, 1.142)
	Male (Reference group = Female)	−0.240	0.049	24.414	<0.001	0.786	(0.715, 0.865)
	Second-tier city (Reference group = Fifth-tier city)	0.541	0.073	55.491	<0.001	1.717	(1.489, 1.980)
	Fourth-tier city (Reference group = Fifth-tier city)	0.000	0.057	0.000	0.995	1.000	(0.895, 1.117)
	High Sleep Diet - Low Activity (Ref. group = High Sleep Diet - High Activity)	−0.027	0.083	0.105	0.745	0.973	(0.826, 1.146)
	Low Sleep Diet - Low Activity (Reference group = High Sleep Diet - High Activity)	0.288	0.054	28.618	<0.001	1.333	(1.200, 1.482)
No anxiety vs. Moderate anxiety	Intercept	−2.853	0.385	55.029	<0.001	—	—
	Age	0.042	0.024	3.062	0.080	1.043	(0.995, 1.093)
	Male (Reference group = Female)	−1.145	0.073	3.936	0.047	0.865	(0.750, 0.998)
	Second-tier city (Reference group = Fifth-tier city)	0.384	0.109	12.541	<0.001	1.469	(1.187, 1.817)
	Fourth-tier city (Reference group = Fifth-tier city)	−0.904	0.084	1.237	0.226	0.910	(0.771, 1.074)
	High Sleep Diet - Low Activity (Ref. group = High Sleep Diet - High Activity)	−0.027	0.083	0.105	0.745	0.973	(0.826, 1.146)
	Low Sleep Diet - Low Activity (Reference group = High Sleep Diet - High Activity)	2.402	0.112	462.291	<0.001	11.044	(8.872, 13.747)
No anxiety vs. Severe anxiety	Intercept	−4.691	0.548	73.299	<0.001	—	—
	Age	0.098	0.034	8.308	0.004	1.103	(1.032, 1.179)
	Male (Reference group = Female)	−0.256	0.101	6.355	0.012	0.774	(0.635, 0.945)
	Second-tier city (Reference group = Fifth-tier city)	0.614	0.140	19.206	<0.001	1.848	(1.404, 2.432)
	Fourth-tier city (Reference group = Fifth-tier city)	−0.087	0.119	0.526	0.468	0.917	(0.726, 1.159)
	High Sleep Diet - Low Activity (Ref. group = High Sleep Diet - High Activity)	−0.128	0.124	1.076	0.300	0.879	(0.690, 1.121)
	Low Sleep Diet - Low Activity (Reference group = High Sleep Diet - High Activity)	2.775	0.139	396.816	<0.001	16.031	(12.201, 21.063)

— indicates no relevant data.

## 4 Discussion

### 4.1 The current situation of anxiety in adolescents and the association between demographic characteristics and anxiety

This study found that 32.62% of adolescents exhibited symptoms of anxiety, highlighting the widespread and serious nature of this issue in the adolescent population (24, 34). Adolescent anxiety often manifests in internalized forms such as persistent worry, fear, and difficulty concentrating. These symptoms are subtle and may be mistakenly viewed as normal developmental changes. However, if left unaddressed, they may escalate into more severe mental health disorders.

Age is significantly positively correlated with both mild and severe anxiety levels. As adolescents grow older, increasing academic demands, interpersonal challenges, and uncertainties about the future may intensify their anxiety (35). A notable gender difference was also observed—female adolescents reported significantly higher anxiety levels than their male counterparts. This disparity may result from a combination of biological factors (e.g., hormonal fluctuations and menstrual cycles) and psychological traits (e.g., heightened emotional sensitivity and stronger stress reactivity) (36, 37). Geographic location also appeared to play a role: adolescents living in second-tier cities reported higher anxiety levels than those in fifth-tier cities. This may be attributed to more intense academic competition, faster urban development, and heightened social expectations in urbanized areas, which can exacerbate psychological stress and negatively impact mental well-being.

### 4.2 Latent class analysis of adolescents’ behavioral lifestyles

This study used LCA to classify adolescents’ behavioral lifestyles and identified three distinct groups: the Low Sleep Diet - Low Activity group, the High Sleep Diet - Low Activity group, and the High Sleep Diet - High Activity group.

Adolescents in the Low Sleep Diet - Low Activity group exhibited the worst performance across all health lifestyle dimensions in this study. This group demonstrated a pattern of insufficient sleep, poor dietary habits, lack of physical activity, and prolonged sedentary behavior on both weekdays and weekends. Insufficient sleep and irregular eating habits may impair the body’s recovery capacity, while a lack of physical activity and prolonged sedentary behavior further limit the effective release of stress, potentially leading to psychological issues such as anxiety and depression (38, 39).

Adolescents in the High Sleep Diet - Low Activity group exhibited relatively favorable patterns in sleep and dietary behaviors but showed clear deficiencies in physical activity and elevated levels of sedentary behavior. Although sufficient sleep and a healthy diet are essential for physical and mental recovery, the absence of regular exercise and prolonged sedentary time may impair their ability to effectively manage stress. Evidence suggests that low activity levels may limit this neurobiological response, thereby increasing susceptibility to anxiety. Moreover, extended sedentary behavior not only reduces energy expenditure but is also strongly associated with mental health risks, including heightened rates of anxiety and depression (40).

The High Sleep Diet–High Activity group exhibited the most balanced and health-enhancing behavioral profile. These adolescents not only maintained a nutritious diet and sufficient sleep but also engaged in high levels of physical activity while minimizing sedentary behavior. Such a multidimensional healthy lifestyle is conducive to improving both physical fitness and psychological resilience, thereby contributing to more favorable physical and mental health outcomes. The characteristics of this group highlight the significance of the synergistic effect of multiple healthy behaviors.

### 4.3 Association between lifestyle and anxiety

This study revealed a significant association between adolescents’ lifestyles and their anxiety levels, with different lifestyle patterns corresponding to varying degrees of anxiety, thereby supporting the research hypothesis.

Adolescents in the Low Sleep Diet–Low Activity group exhibited higher rates of mild, moderate, and severe anxiety symptoms. From the perspective of self-regulation theory, individuals rely on internal physiological and psychological resources to manage external stressors and maintain emotional stability and behavioral control (41). However, insufficient sleep can impair the nervous system’s ability to repair and regulate itself, thereby weakening emotional regulation (42). Poor dietary habits may disrupt neurochemical synthesis and brain function, undermining mood stability (43). Furthermore, a chronic lack of physical activity reduces the secretion of key neurotransmitters such as dopamine and serotonin, which are essential for emotional resilience and stress recovery (44, 45). These unhealthy lifestyle factors not only pose individual risks for anxiety but may also interact synergistically to exacerbate emotional dysregulation.

Adolescents in the High Sleep Diet–Low Activity group exhibited significantly higher levels of mild anxiety compared to those in the High Sleep Diet–High Activity group. Although this group maintained adequate sleep and healthy dietary habits, their limited participation in physical activity reduced their capacity to regulate emotions and relieve stress through exercise. The lack of effective outlets for emotional release may contribute to the accumulation of anxiety and the formation of a negative emotional cycle. In addition, this group demonstrated elevated levels of sedentary behavior, increasing the risk of overweight and chronic health conditions. Obesity, in turn, has been closely associated with mental health issues such as anxiety and depression (40, 46). While the group shows strengths in certain health behaviors, insufficient physical activity may still exert a significant adverse impact on their mental well-being.

In contrast, adolescents in the High Sleep Diet - High Activity group exhibited the lowest levels of anxiety, According to the Health Belief Model (HBM), individuals are more likely to adopt positive health behaviors when they recognize the severity of potential health threats and clearly perceive the benefits of preventive actions (47). Adolescents in the High Sleep Diet–High Activity group may possess a heightened awareness of the mental health benefits associated with sufficient sleep, balanced nutrition, and regular physical activity. This perceived benefit may motivate them to develop and maintain healthy lifestyle habits, thereby reducing their risk of anxiety and enhancing emotional resilience (48, 49).

### 4.4 Implications of the findings

Our findings have important practical implications. They further validate the influence of lifestyle on adolescent anxiety and offer new perspectives and directions for the prevention and intervention of anxiety among youth. The results provide strong support for existing mental health intervention theories and underscore the profound impact of individual health behaviors on psychological well-being. Schools, families, and society must collaborate to guide adolescents in adopting healthy lifestyles, preventing and alleviating anxiety, and promoting their physical, and mental health, and overall development. Schools can implement health education and behavioral interventions to help students improve unhealthy lifestyle habits, particularly in areas such as sleep and physical activity. Parents should focus on fostering a supportive family environment and encourage healthy routines in diet, rest, and exercise. Meanwhile, society should provide greater access to mental health resources to help adolescents enhance their mental health literacy.

### 4.5 Research strengths and limitations

One strength of the current study lies in its investigation of the relationship between adolescents’ lifestyles and anxiety, providing valuable data to inform future mental health interventions and lifestyle modifications. Furthermore, by applying LCA, this study identified distinct patterns of adolescent lifestyles and explored the complex associations between these patterns and anxiety, thereby challenging overly simplistic perspectives. The study also offers targeted policy recommendations for society, families, and schools, highlighting strategies to promote healthy lifestyles as a means of alleviating adolescent anxiety symptoms.

However, this study has several limitations. First, due to its cross-sectional design, it is unable to reveal dynamic relationships among variables or draw causal inferences. Future research should adopt longitudinal designs and incorporate methods such as network analysis to systematically explore the mechanisms linking lifestyle and anxiety, as well as to clarify their temporal development. Second, although the study had a large sample size, all participants were recruited from a single city in northern China, which limits the generalizability of the findings. Future studies should include adolescents from more provinces and other countries to enhance the applicability and universality of the results.

## 5 Conclusion

The prevalence of anxiety among adolescents was 32.62%. Adolescents’ behavioral lifestyles can be classified into three main categories: the High Sleep Diet-Low Activity group, the High Sleep Diet-High Activity group, and the Low Sleep Diet-Low Activity group. Anxiety symptoms were influenced by various factors, including age, gender, school location, and behavioral lifestyle. The Low Sleep Diet-Low Activity group exhibited the highest levels of anxiety (mild, moderate, and severe anxiety), while the High Diet Sleep-Low Activity group was more likely to experience mild anxiety. In contrast, the High Sleep Diet-High Activity group demonstrated the lowest anxiety levels. This study are of significant importance for understanding the mechanisms behind adolescent anxiety, revealing a close association between adolescent anxiety and comprehensive behavioral lifestyles. This finding fills a gap in the existing literature regarding the impact of different integrated lifestyle patterns on adolescent mental health. Future research could further investigate the specific mechanisms through which different lifestyle patterns influence adolescent anxiety, and explore in greater depth how lifestyle modifications may help alleviate anxiety symptoms in this population. In addition, future studies may extend to populations across diverse cultural backgrounds and age groups to examine the generalizability and universality of the relationship between lifestyle and anxiety.

## Data Availability

The raw data supporting the conclusions of this article will be made available by the authors, without undue reservation.
